# Using imputed whole-genome sequence data to improve the accuracy of genomic prediction for parasite resistance in Australian sheep

**DOI:** 10.1186/s12711-019-0476-4

**Published:** 2019-06-26

**Authors:** Mohammad Al Kalaldeh, John Gibson, Naomi Duijvesteijn, Hans D. Daetwyler, Iona MacLeod, Nasir Moghaddar, Sang Hong Lee, Julius H. J. van der Werf

**Affiliations:** 1 Cooperative Research Centre for Sheep Industry Innovation, Armidale, NSW 2351 Australia; 2 School of Environmental and Rural Science, University of New England, Armidale, NSW 2351 Australia; 3 Centre for AgriBioscience, Agriculture Victoria, Bundoora, VIC 3083 Australia; 4 School of Applied Systems Biology, La Trobe University, Bundoora, VIC 3083 Australia; 5 Australian Centre for Precision Health, University of South Australia Cancer Research Institute, University of South Australia, Adelaide, SA 5000 Australia

## Abstract

**Background:**

This study aimed at (1) comparing the accuracies of genomic prediction for parasite resistance in sheep based on whole-genome sequence (WGS) data to those based on 50k and high-density (HD) single nucleotide polymorphism (SNP) panels; (2) investigating whether the use of variants within quantitative trait loci (QTL) regions that were selected from regional heritability mapping (RHM) in an independent dataset improved the accuracy more than variants selected from genome-wide association studies (GWAS); and (3) comparing the prediction accuracies between variants selected from WGS data to variants selected from the HD SNP panel.

**Results:**

The accuracy of genomic prediction improved marginally from 0.16 ± 0.02 and 0.18 ± 0.01 when using all the variants from 50k and HD genotypes, respectively, to 0.19 ± 0.01 when using all the variants from WGS data. Fitting a GRM from the selected variants alongside a GRM from the 50k SNP genotypes improved the prediction accuracy substantially compared to fitting the 50k SNP genotypes alone. The gain in prediction accuracy was slightly more pronounced when variants were selected from WGS data compared to when variants were selected from the HD panel. When sequence variants that passed the GWAS $- log_{10} (p\,value)$ threshold of 3 across the entire genome were selected, the prediction accuracy improved by 5% (up to 0.21 ± 0.01), whereas when selection was limited to sequence variants that passed the same GWAS $- log_{10} (p\,value)$ threshold of 3 in regions identified by RHM, the accuracy improved by 9% (up to 0.25 ± 0.01).

**Conclusions:**

Our results show that through careful selection of sequence variants from the QTL regions, the accuracy of genomic prediction for parasite resistance in sheep can be improved. These findings have important implications for genomic prediction in sheep.

## Background

Traditionally, genetic improvement in livestock species has relied on the use of phenotypes and pedigree information of animals to predict their breeding values. This approach has resulted in substantial genetic gains for most production traits. However, the efficiency of these methods declines for traits that are difficult, or expensive to measure, or have a low heritability. Gastrointestinal parasites pose a major health and economic burden to the Australian sheep industry with an estimated annual cost of $436 million [1]. Breeding sheep for enhanced resistance provides a sustainable long-term solution for controlling infections.

Genomic selection is increasingly applied in breeding programs, offering an alternative to conventional methods; it can potentially increase the rates of genetic gain [2] and could be particularly useful for traits that are difficult to improve using traditional methods. Whole-genome sequence (WGS) data can potentially accelerate genetic improvement by including causal mutations or variants in strong linkage disequilibrium (LD) with causal mutations. However, in practice the use of all the variants from WGS data in cattle populations resulted in little to no improvement in the accuracy of genomic predictions when investigated within breed [3, 4]. Even if all the causal variants are included in the sequence data, the number of causal variants that underlie quantitative trait loci (QTL) represent only a small proportion, with the majority of these sequence variants being trait neutral. Unless only the markers in strong LD with causal mutations and multi-breed datasets are used for genomic prediction, the accuracy of prediction might not increase, since prediction might be encumbered by the large number of uninformative markers. Furthermore, the effective population size (Ne) is typically small to moderate in some livestock populations such as Holstein dairy cattle [5]. For small values of Ne, increasing marker density will have a limited impact on the accuracy of genomic predictions [6].

Previous studies on genomic prediction often gave equal weight to evenly spaced markers, whereas studies now increasingly place more emphasis on quantitative trait loci (QTL) or variants that are selected from genome-wide association studies (GWAS). A larger increase in prediction accuracy has been reported in dairy cattle when sequence variants that are selected from GWAS were treated as a separate component alongside the 50k data compared to when the 50k or WGS data were fitted alone (e.g. [4, 7, 8]). A simulation study by [9] showed that sequence data can potentially improve genomic prediction by using only the variants that were close to causal mutations, whereas the accuracy dropped quickly when the distance between causal mutations and markers increased.

Most existing variant selection methods are based on GWAS results [4, 7, 8]. However, false positive and false negative associations can complicate variant selection, likely limiting improvements in prediction accuracy. Most GWAS appear to be relatively underpowered due to the small effects of causal variants and to limited sample size. Optimizing the power of GWAS is both crucial and challenging. Without increasing sample size, power could be increased by combining independent GWAS studies together, followed by an integrative meta-analysis [10–12]. Another strategy to increase the power of GWAS is to develop statistical methods that can capture the genetic variation of the trait in genomic regions versus single loci. Regional heritability mapping (RHM) offers an alternative to conventional GWAS methods by integrating effects of common and rare variants within one genomic region [13]. Thus, RHM may identify QTL that may otherwise go undetected by conventional GWAS methods, potentially improving the prediction accuracy.

The objectives of this study were to: (1) compare the accuracies of genomic prediction for parasite resistance based on WGS data to those based on 50k and HD SNP panels; (2) investigate whether the use of variants in QTL regions that were selected from WGS data would improve the accuracy more than variants selected from the HD SNP panel; and (3) compare the prediction accuracies between variants selected from RHM, GWAS, or GWAS in genomic regions detected by RHM. To evaluate the effect of preselected variants on improving prediction accuracy, we used a model in which a genomic relationship matrix (GRM) from the 50k SNP genotypes was fitted alongside a GRM from the preselected variants. We hypothesized that this would increase prediction accuracy for parasite resistance compared to fitting the 50k SNP genotypes alone.

## Methods

### Phenotypes and population structure

**Fig. 1:**
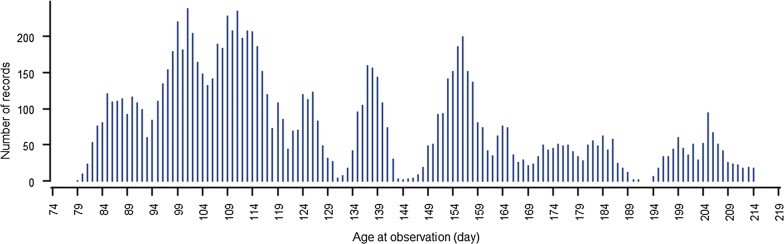
Number of records according to age (in days)

Parasite resistance, as measured by worm egg counts (WEC), was investigated in a large multi-breed sheep population from the information nucleus (IN) flock of the Australian Cooperative Research Centre for Sheep Industry Innovation (CRC). Details on the IN flock, design, and trait collection and measurements are described in van der Werf et al. [14]. The sheep CRC has developed a standardized procedure for collecting and measuring this trait. When a random faecal sample within a management group exceeded a threshold of 1000 eggs per gram (epg) in sites predominated by *Haemonchus contortus*, and 500 epg in sites predominated by other species, faecal samples were collected from all individuals. Worm eggs were then counted using a modified McMaster counting technique [15], and the presence of three main strongyle species, i.e. *H. contortus*, *T. colubriformis*, and *T. circumcincta* was determined. A total of 10,950 animals were included in the analysis. Ages ranged from 79 to 214 days with an average of 130 days (Fig. 1). The animals descended from 612 sires and 6639 dams.

Various breeds were represented in the population with Merino reaching the most significant proportion (64.6%), and only individuals from this breed had a substantial proportion of purebred animals (39%). The remaining breeds were mainly represented by crossbred offspring of Border Leicester (BL), Poll Dorset (PD) and White Suffolk (WS) sires and Merino or Border Leicester × Merino ewes. Breed group size ranged from 4262 sheep for purebred Merino to 99 for East Friesian × Merino cross. The complete breed content of this population is in Table 1.

**Table 1:** Average proportions of different breeds in the dataset

Breed	BL	COR	COOP	EF	WD	PD	TEX	DR	AF	SF	WS	PS	MER
Proportion (%)	10.9	0.8	6.7	0.5	0.8	6.7	1.7	0.5	1.3	1.6	2.9	0.9	64.6

*BL* Border Leicester, *COR* Corriedale, *COOP* Coopworth, *SF* Suffolk, *WS* White Suffolk, *EF* East Friesian, *WD* White Dorper, *PD* Poll Dorset, *TEX* Texel, *AF* Australian Finnsheep, *DR* Dorper, *PS* Prime Samm, *MER* Merino

### Genotypes and quality control

#### 50k genotypes

All animals with WEC phenotypes were genotyped with the Illumina 50k SNP panel (Illumina Inc., San Diego, CA, USA). The following quality measures were applied to the 50k SNP data: SNPs were removed if they had a minor allele frequency (MAF) lower than 0.01, a call rate lower than 90%, an Illumina GenCall score (GC) less than 0.6, if they were not in Hardy–Weinberg equilibrium (a *p* value cut-off of $10^{ - 15}$), and if the heterozygosity rate for each SNP deviated more than 3 standard deviations (SD) from the population mean. SNPs located on the X and Y chromosomes were also excluded. Furthermore, an individual sample for which the correlation between its genotype and that of another sample was equal or greater than 0.99 was removed. Missing genotypes were imputed using Beagle [16]. After applying the quality control measures, 48,599 SNPs were retained.

#### High density (HD) and whole-genome sequence (WGS) genotypes

All animals with WEC phenotypes were then imputed from 50k genotypes to the 600 kOvine Infinium^®^ HD SNP BeadChip panel (International Sheep Genomic Consortium and FarmIQ Project NZ) and then to whole-genome sequence (WGS). The details of the imputation to WGS for sheep CRC animals (including those used here with WEC phenotypes) are in Bolormaa et al. [17], but it is briefly described here for completeness.

The high-density (HD) genotypes were imputed using a reference set of 1881 animals with real HD, which represented four main breeds (Merino, Poll Dorset, Border Leicester, and White Suffolk): 1042 represented various crosses of these breeds, whereas purebreds included 677 Merino, 47 White Suffolk, 44 Poll Dorset, 32 Border Leicester, and 39 from 10 other breeds. After applying the same quality controls as above, 510,065 SNPs were retained, and these 1881 HD animals were used as a reference set to impute the 50k genotypes to HD using Minimac3 [18]. Prior to imputation, phasing was performed on both the 50k-genotyped and HD-genotyped animals separately using Eagle2 [19]. The accuracy of imputation to HD, tested within subsets of animals with observed HD genotypes, was on average high (0.98).

The phenotyped animals were then imputed from HD SNP genotypes to WGS, again with the combination of Eagle and Minimac3 [18, 19]. The reference population with WGS included 376 animals from the main Australian breeds that were sequenced (with ~ 10 × coverage) by the sheep CRC (some were immediate ancestors of the animals that were phenotyped for this study). Data on those animals were combined with WGS data that were available on 350 animals of European breeds from the “Sheep Genomes DB” project [20]. Details of the breed composition of the WGS reference animals are in Bolormaa et al. [17]. Sequence variants were imputed for all ovine autosomes but not for the X and Y chromosomes. All variants with a Minimac3 imputation quality statistic (R2) higher than 0.4 were retained, resulting in a final set of 31,154,082 imputed variants. The mean accuracy of imputation from HD to WGS for this filtered set of variants in Merinos, Poll Dorset or Merino × Bolder Leicesters was estimated to be 0.97 [17].

### Statistical models

#### Experimental design

To avoid potential bias in estimating breeding values, the whole dataset was split randomly into a QTL discovery set and a training/validation set while retaining the same proportion of breeds in each set. The QTL discovery subset consisted of 6431 individuals, whereas the remaining 4500 individuals were assigned to the training/validation subset. Validation groups were chosen from the training/validation subset using a tenfold cross-validation design. The performance of genomic prediction was evaluated across sire families, i.e. entire sire families in the training/validation subset were randomly chosen and combined into ten non-overlapping subsets, each with roughly 450 animals. One of the ten subsets served as the validation group while all the remaining subsets served as the training population. The observed phenotypes of the validation animals were then masked and genomic breeding values were estimated. The accuracy of genomic prediction was calculated as the correlation between the estimated genomic breeding values (GEBV) of the validation set and their observed phenotypes, divided by the square root of the heritability, which was estimated based on the phenotypes of all the animals. We calculated the average correlation across the ten folds of the CV and the entire tenfold CV was replicated ten times and average accuracies were calculated across the replicate tenfold CV. Furthermore, the regression coefficient of the observed phenotypes on GEBV was calculated to evaluate the bias of genomic predictions and those were averaged across the replicate tenfold CV.

#### Association studies using sequence information

Variant selection was based on the GWAS that was performed on the QTL discovery subset, whereas effects of the selected variants were estimated and validated using the training/validation subset. GWAS and genome-wide RHM were performed on the QTL discovery subset using WGS data. Phenotypes were pre-adjusted for fixed effects before being used in GWAS and RHM. The fixed effects included in the model to pre-correct phenotypes were: age of the animal at WEC recording, age of dam, gender, rearing type × birth type, contemporary groups (combination of flock site, birth year and management group effects) and breed proportions, which were fitted as fixed covariates. For GWAS, each variant was fitted separately, and a pedigree relationship matrix was fitted to account for population and pedigree structure. A linear mixed model was performed using the GEMMA software [21] as follows:

1 $${\mathbf{y}}^{*} = 1\mu + {\mathbf{W}}_{\varvec{i}} g_{i} + {\mathbf{Za}} + {\mathbf{e}},$$

where ${\mathbf{y}}^{*}$ is a vector of adjusted phenotypes (residuals), $\varvec{ }\mu$ is the overall mean, ${\mathbf{W}}_{{\mathbf{i}}}$ is a vector of genotypes for SNP $i$ (coded as 0, 1, or 2 for the genotypes 00, 01/10, or 11, respectively), $g_{i}$ is the size of the effect of the marker (allele substitution effect), ${\mathbf{Z}}$ is a design matrix for the random additive genetic effects; ${\mathbf{a}}$ is a vector of random additive genetic effects assumed to be distributed as $\sim\,N\left( {0,{\mathbf{A}}\sigma_{a}^{2} } \right)$, where ${\mathbf{A}}$ is the numerator relationship matrix calculated from pedigree records extending across nine generations using the pedigree package in R [22], and ${\mathbf{e}}$ is the vector of residuals. SNP effects were estimated, and a Wald test was performed to calculate the *p* values of each SNP effect.

Regional heritability mapping (RHM) analysis was performed on WGS data using MTG2 [23] and using the same GWAS subset as that for the association study. For RHM, each chromosome was divided into regions with a pre-defined number of SNPs and the additive genetic variance attributable to the joint SNP effects within each window was estimated. A window size of 12,000 SNPs (~ 1 Mbp) was used to construct the genomic relationship matrix (${\mathbf{GRM}}$) from WGS genotypes in that specific region and the window was then shifted along the whole genome in steps of 6000 SNPs (0.5 Mbp). The significance of each window was then assessed by the likelihood ratio test (LRT), comparing the full model (Model 2), which includes the regional effect, with the base model which includes mean, and random animal and error terms (Model 3). Variance components were estimated using the residual maximum likelihood (REML) analysis as implemented in MTG2 assuming the following models:

2 $${\mathbf{y}}^{*} = 1\mu + {\mathbf{Z}}_{i} u_{i} + {\mathbf{Za}} + {\mathbf{e}} ,$$

3 $${\mathbf{y}}^{*} = 1\mu + {\mathbf{Za}} + {\mathbf{e}} ,$$

where the terms are as defined above in Model 1, but where $u_{i}$ is the additive genetic effect estimated from SNPs in region $i$ (window) and assumed to be distributed as $N\left( {0,{\mathbf{GRM}}_{i} \sigma_{{u_{i} }}^{2} } \right)$, where ${\mathbf{GRM}}_{i}$ is the genomic relationship matrix constructed from SNPs in region $i$, and $\sigma_{{u_{i} }}^{2}$ is the genetic variance explained by SNPs in region $i$. The phenotypic variance ($\sigma_{p}^{2}$) was given by $\sigma_{{u_{i} }}^{2} + \sigma_{a}^{2} + \sigma_{e}^{2}$ and therefore the regional genomic heritability was estimated as ${\text{h}}_{{u_{i} }}^{2} = \sigma_{{u_{i} }}^{2} /\sigma_{p}^{2}$.

For the RHM approach, LRT was assumed to follow a mixture of $0.5\chi_{\left( 1 \right)}^{2}$ and $0.5\chi_{(0)}^{2}$ distributions [13]. In total, 4659 windows were tested across the genome. Windows identified with $- log_{10} \left( p \right)$ higher than 3 were selected for further refinement using RHM analysis with a smaller 250-kbp window size and using variants within each window from either the 50k, HD, or WGS data in order to compare mapping precision across the three marker densities.

### Variant selection

Seven scenarios with different subsets of prediction variants were evaluated, using either WGS or HD genotypes and variants that were selected based on either GWAS, RHM or a combination of both. An overview of all scenarios, including the number of selected variants in each scenario, is in Table 3. In scenarios 1 and 2, variants were selected from GWAS results, testing all the variants across the entire genome. Variants that passed the GWAS $- log_{10}$ (*p* value) threshold of 3 were selected. In scenarios 3 to 5, variants were selected from windows that were identified by RHM analysis using a window size of 1 Mbp for scenario 3 and 250 kbp for scenarios 4 to 5. Windows that passed the $- log_{10} \left( p \right)$ threshold of 3 were considered for variant selection, and all variants within those windows were selected. In scenarios 6 and 7, variants were also selected from windows that passed the RHM $- log_{10} \left( p \right)$ threshold of 3, however variant selection in those windows was limited to variants with a GWAS $- log_{10} \left( p \right)$ higher than 3. For all the scenarios, the selected variants were pruned for high LD ($r^{2} \ge 0.95$) using PLINK (http://pngu.mgh.harvard.edu/purcell/plink) on 100-kbp windows by shifting every 50 kbp.

### Genomic prediction

Genomic estimated breeding values (GEBV) were calculated using the genomic best linear unbiased prediction (GBLUP) model as implemented in MTG2 [23]. MTG2 provides REML analysis to estimate variance components and breeding values. GBLUP was performed using a ${\mathbf{GRM}}$ built from the 50k, HD, or WGS markers. A GBLUP model that includes both a ${\mathbf{GRM}}$ from the 50k data (${\mathbf{GRM}}_{50K}$) fitted together with a ${\mathbf{GRM}}$ from the selected variants (${\mathbf{GRM}}_{top}$) was also evaluated. Variants used in ${\mathbf{GRM}}_{top}$ were selected from one of the seven scenarios in Table 3. Variants included in ${\mathbf{GRM}}_{top}$ were excluded from the ${\mathbf{GRM}}_{50K}$ for that scenario. In the models where ${\mathbf{GRM}}_{top}$ was fitted together with ${\mathbf{GRM}}_{50K}$, GEBV were computed by adding the estimated genetic effects from ${\mathbf{GRM}}_{top}$ to those estimated from ${\mathbf{GRM}}_{50K}$.

### Variance explained by the selected variants

In the scenarios in which ${\mathbf{GRM}}_{top}$ was tested, the proportion of variance explained by the selected variants $h_{top}^{2}$ was calculated as:

$$h_{top}^{2} = \frac{{\sigma_{top}^{2} }}{{\sigma_{top}^{2} + \sigma_{50k}^{2} + \sigma_{e}^{2} }},$$

where $\sigma_{top}^{2}$ is the variance explained by the selected variants in each scenario and $\sigma_{50k}^{2}$ is the variance explained by the 50k SNPs.

## Results

### Association analyses

**Fig. 2:**
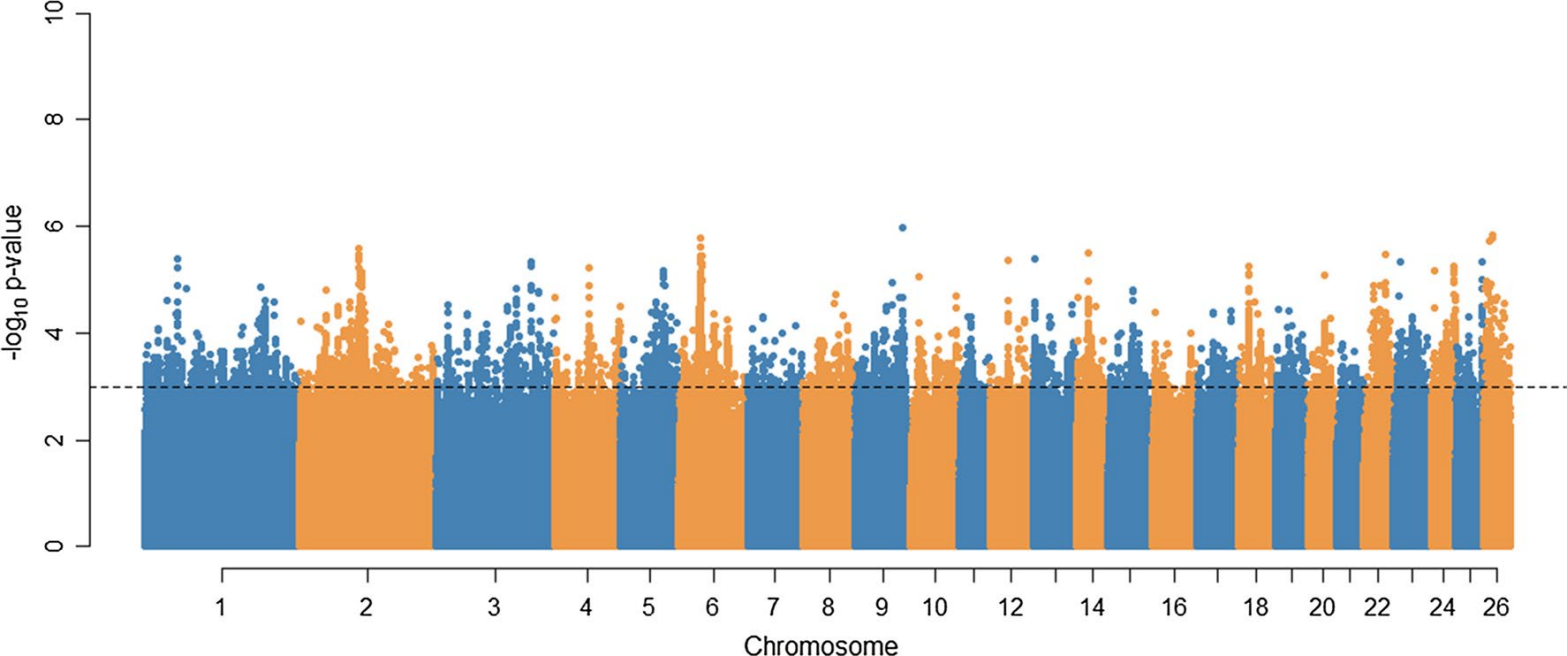
Manhattan plot of GWAS results for parasite resistance in sheep. The y-axis shows the $- log_{10} (p)$ value for each marker and the x-axis shows the physical position of each marker across the genome. The dashed horizontal line corresponds to the $- log_{10}$ (*p* value) threshold of 3

**Fig. 3:**
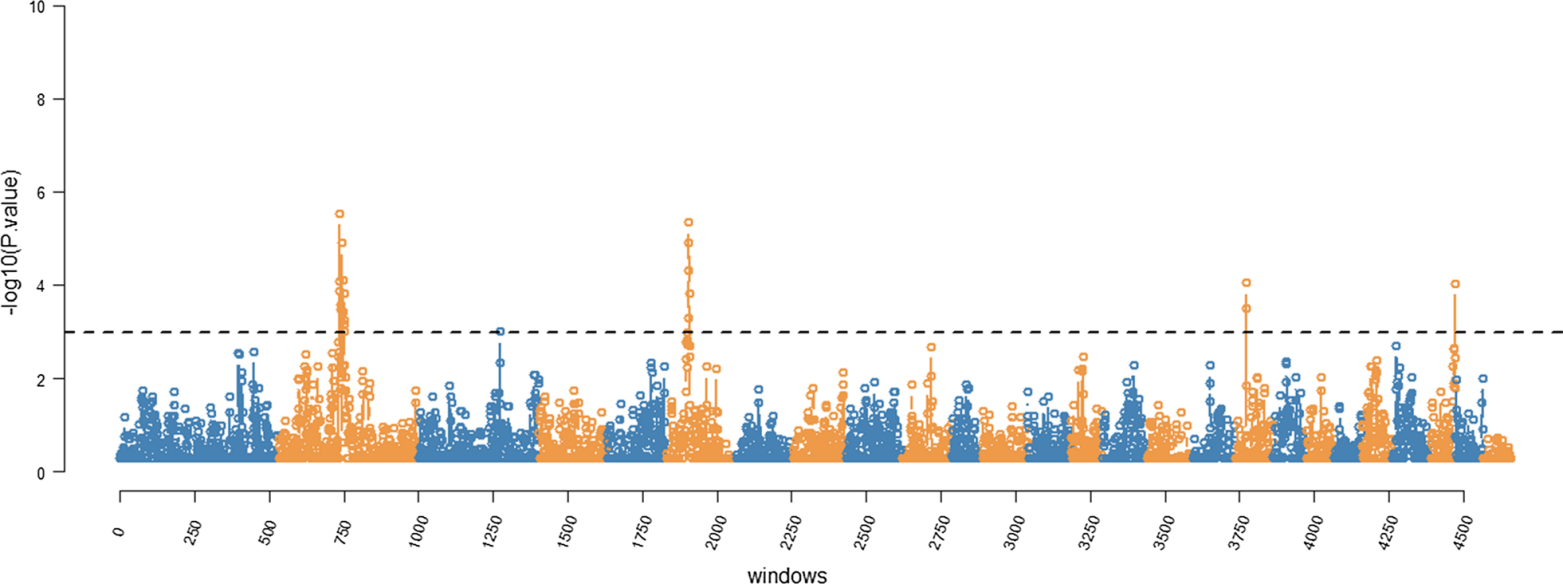
Manhattan plot of RHM results for parasite resistance in sheep. The y-axis shows the $- log_{10}$ (*p* values) for each window and the x-axis shows the window number across the genome. The dashed horizontal line corresponds to the $- log_{10}$ (*p* value) threshold of 3

Manhattan plots of the GWAS and RHM results for parasite resistance using WGS data are in Figs. 2 and 3, respectively. The numbers of variants from GWAS with a $- log_{10} \left( p \right)$ higher than 3 before and after LD pruning were equal to 17,154 and 3913, respectively, all of these spanning the 26 ovine autosomes. For RHM based on WGS data, only 11 windows, which overlapped with five regions on OAR2, 3, 6, 18, and 24 passed the $- log_{10} \left( p \right)$ threshold of 3. These five RHM regions, between 105.2 and 119.3 Mbp on OAR2, 148 and 149 Mbp on OAR3, 34.3 and 38.2 Mbp on OAR6, 17.2 and 18.3 Mbp on OAR18, and 39.9 and 41.9 Mbp on OAR24, all contained SNPs that were also selected in the GWAS results at the $- log_{10} \left( p \right)$ thresholds 3.

**Fig. 4:**
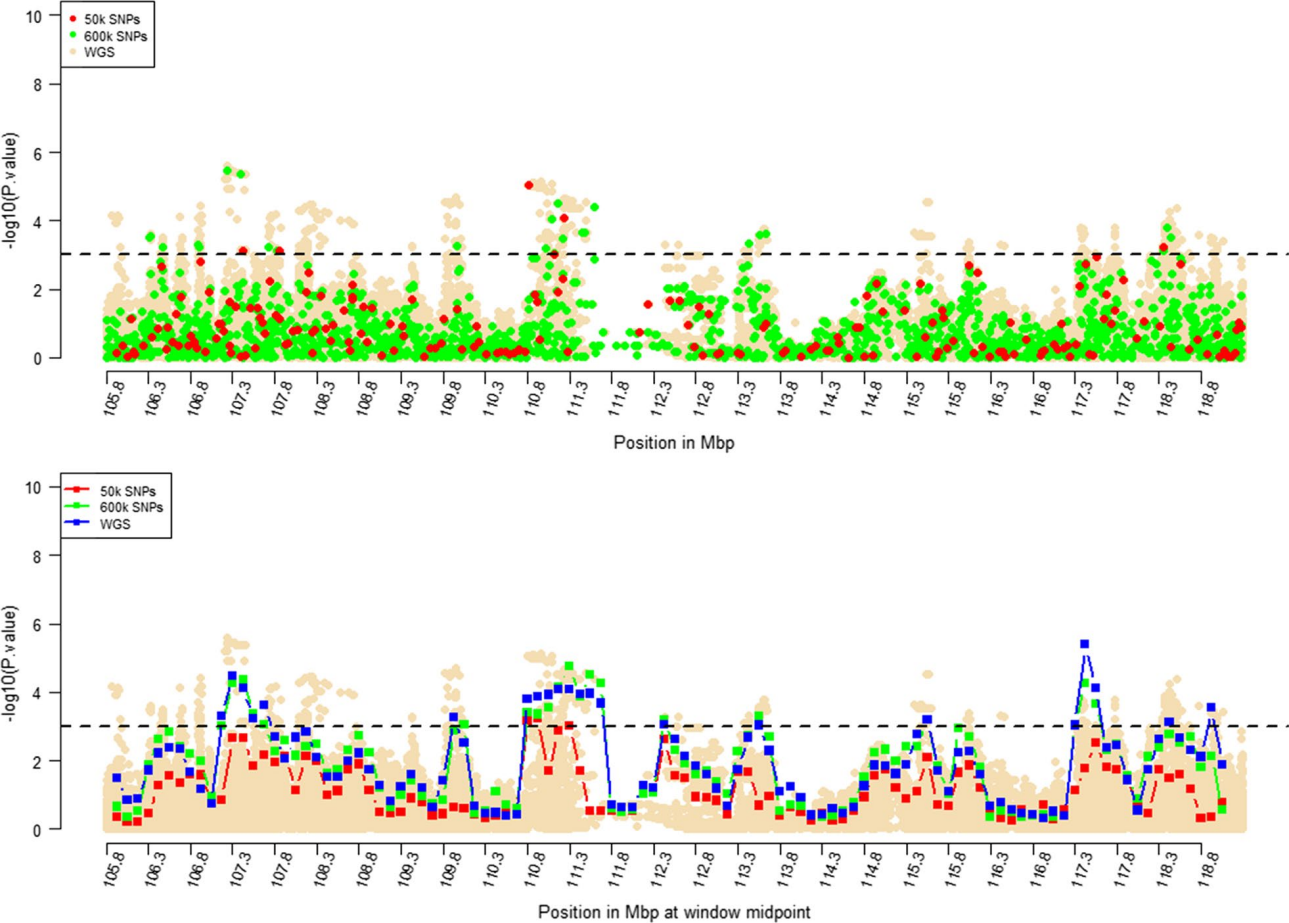
GWAS (top plot) and RHM (bottom plot) results for the OAR2 region between 105 and 119 Mbp using three marker densities: 50k, HD, and WGS. Each window was positioned at its midpoint. Wheat-coloured dots are GWAS results from WGS variants

**Fig. 5:**
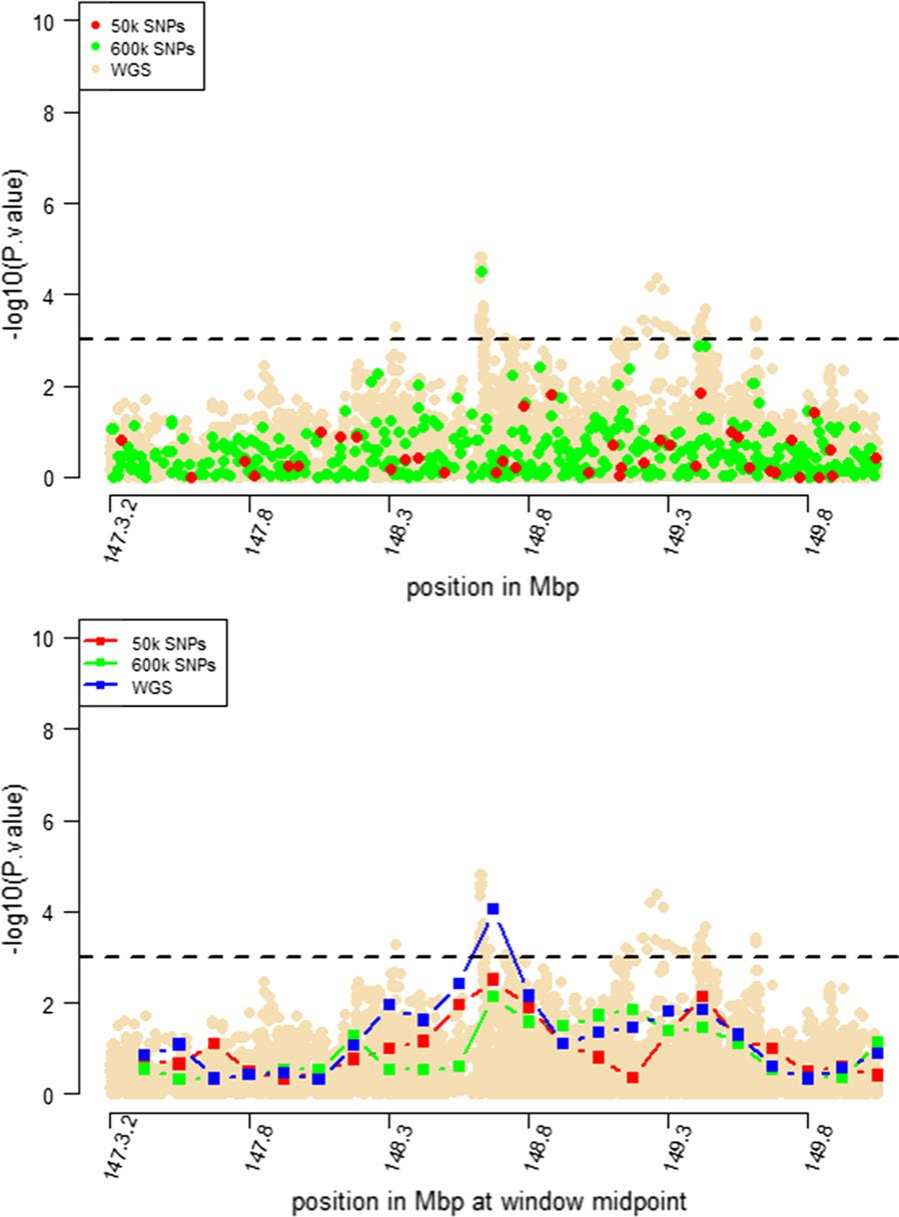
GWAS (top plot) and RHM (bottom plot) results for the OAR3 region between 147.3 and 148.8 Mbp using three marker densities: 50k, HD, and WGS. Each RHM result was positioned at its midpoint. Wheat-coloured dots are GWAS results from WGS variants

**Fig. 6:**
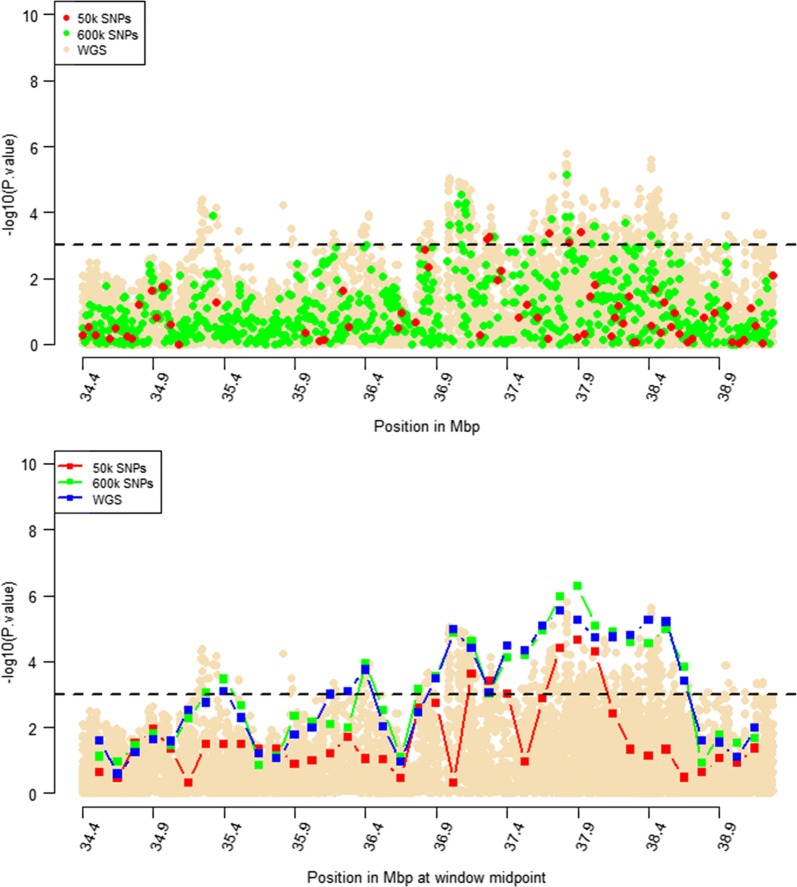
GWAS (top plot) and RHM (bottom plot) results for the OAR6 region between 34.4 and 36.9 Mbp using three marker densities: 50k, HD, and WGS. Each RHM result was positioned at its midpoint. Wheat-coloured dots are GWAS results from WGS variants

**Fig. 7:**
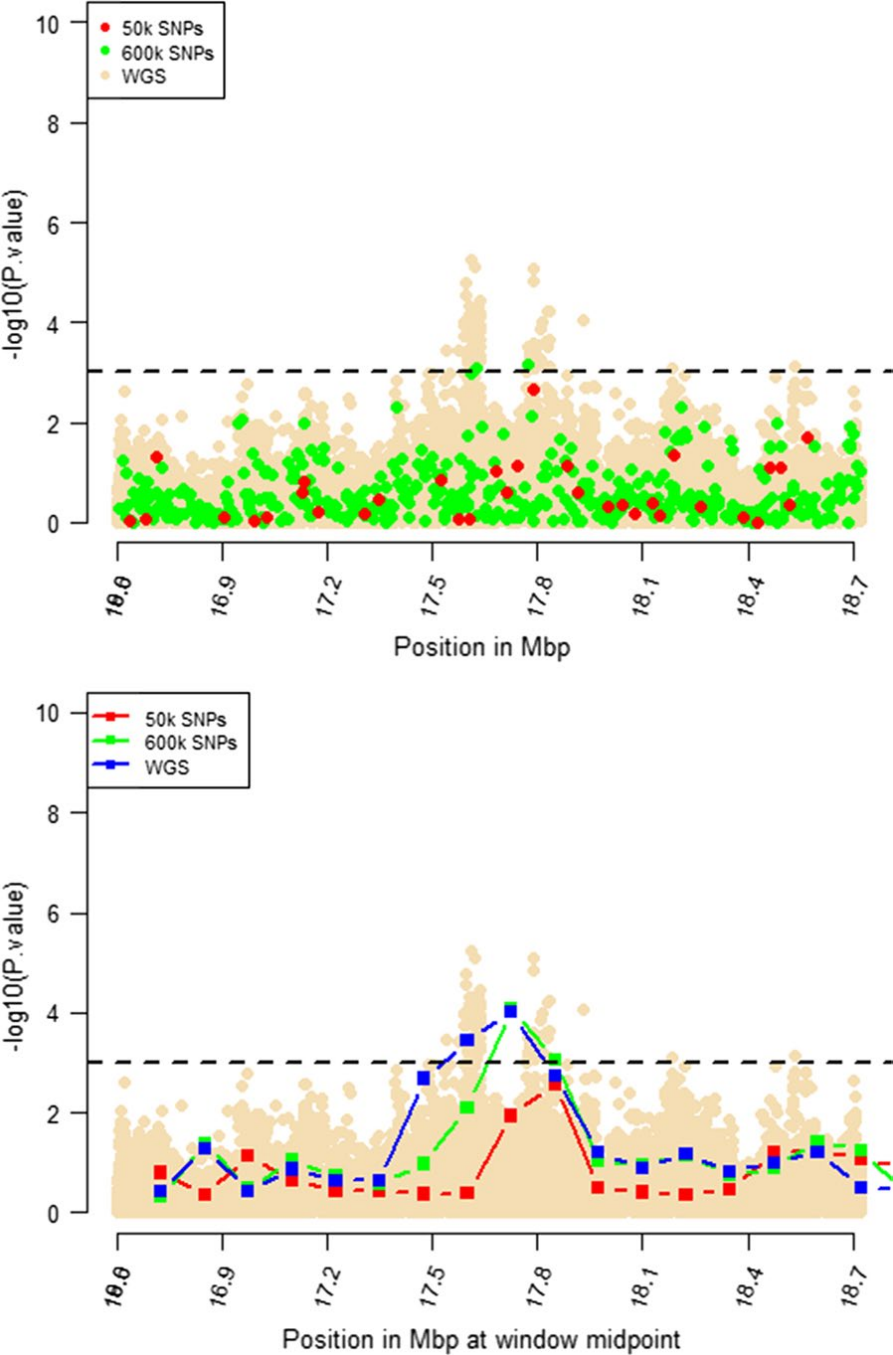
GWAS (top plot) and RHM (bottom plot) results for the OAR18 region between 16.8 and 18.7 Mbp using three marker densities: 50k, HD, and WGS. Each RHM result was positioned at its midpoint. Wheat-coloured dots are GWAS results from WGS variants

**Fig. 8:**
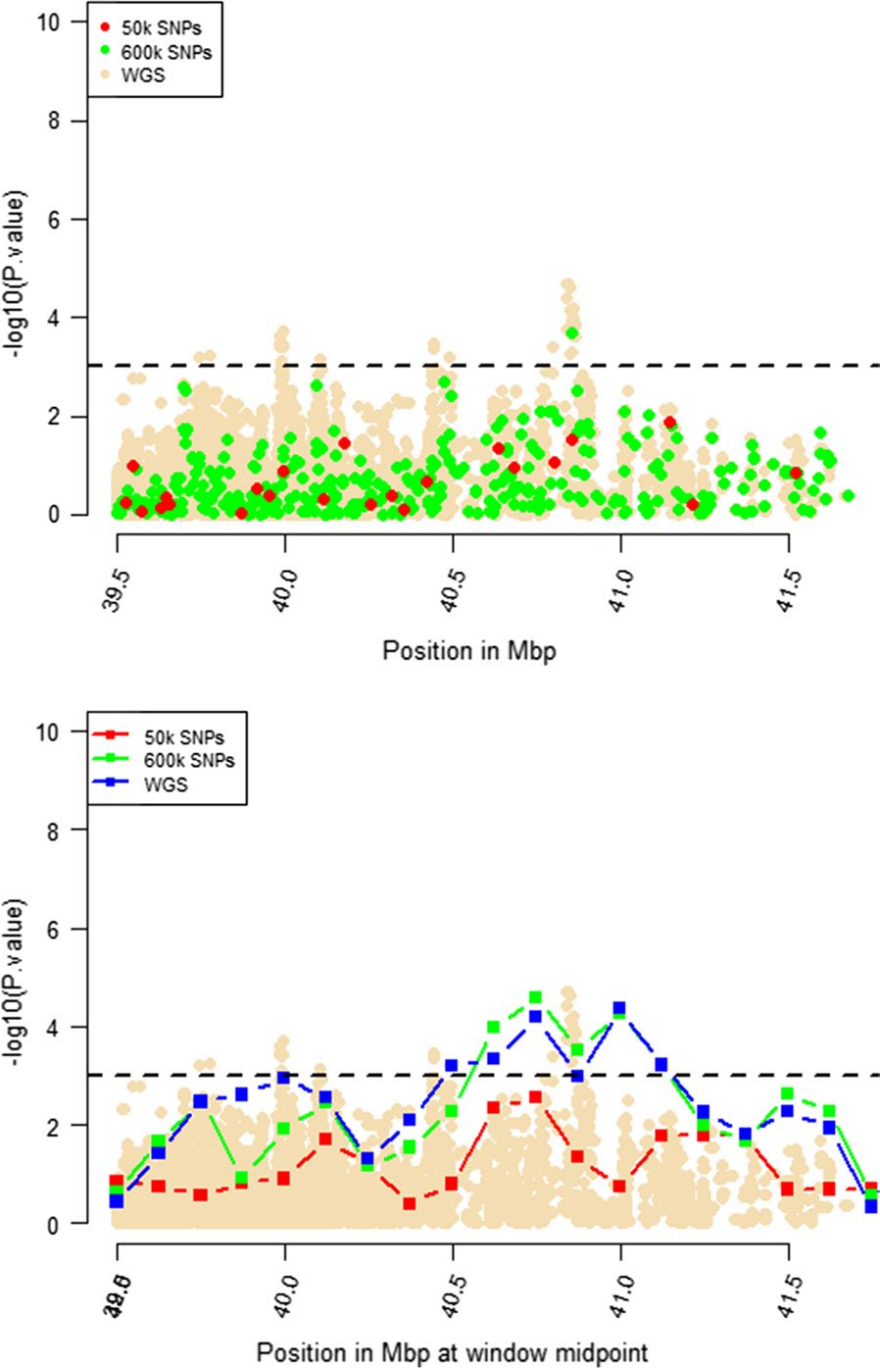
GWAS (top plot) and RHM (bottom plot) results for the OAR24 region between 39.6 and 41.5 Mbp using three marker densities: 50k, HD, and WGS. Each RHM result was positioned at its midpoint. Wheat-coloured dots are GWAS results from WGS variants

Windows that were identified by RHM using WGS data with $- log_{10} \left( p \right)$ higher than 3 were selected for further RHM analysis using a 250-kbp window size. To investigate the impact of marker density on mapping precision, RHM was performed on those regions and using variants from the three marker densities: 50k, HD, and whole-genome sequence variants. *P* values of RHM using a 250-kbp window size from the HD data were similar to those obtained from sequence data for the regions on OAR2, 6, 18, and 24, except for the region on OAR3, which had a higher *p* value when RHM was based on sequence data compared to HD data (Figs. 4, 5, 6, 7, 8). However, *p* values of RHM based on the 50k panel were lower than those of RHM based on the HD and WGS panels. GWAS results for the selected regions based on WGS variants generally have both increasingly higher and sharper peaks than GWAS results based on HD or 50k variants.

### Genomic prediction using the three marker densities

Accuracies of genomic predictions for parasite resistance based on GBLUP using all genome-wide variants from the three marker densities (50k, HD, and WGS) are in Table 2. Using all the variants from WGS and HD data gave a 3% ($0.19 \pm 0.01$) and 2% ($0.18 \pm 0.01$) increase in prediction accuracy, respectively, compared to using variants from the 50k panel ($0.16 \pm 0.02$). The slope of the regression of the adjusted phenotypes on GEBV, improved from 0.82 ± 0.015 when using the 50k variants to 0.91 ± 0.014 and 0.95 ± 0.013 when using the HD and WGS variants, respectively.

**Table 2:** Accuracy of genomic prediction and regression coefficient (slope) of adjusted phenotypes on GEBV using different marker panels (50k, HD, and WGS)

Marker panel	Accuracy	Slope
50k	0.16 ± 0.02	0.82 ± 0.02
HD	0.18 ± 0.01	0.91 ± 0.01
WGS	0.19 ± 0.01	0.95 ± 0.01

### Genomic prediction using selected variants

Accuracies of genomic predictions using variants that were selected from either HD or WGS data fitted together with the 50k variants are in Table 3. Fitting a GRM from the selected variants with a GRM from the 50k genotypes improved the prediction accuracy by varying degrees when compared to fitting the 50k SNPs alone. Sequence variants selected from RHM window sizes of 250kbp (scenario 4) slightly improved the prediction accuracy by 1% compared to sequence variants selected from RHM window sizes of 1 Mbp (scenario 3).

**Table 3:** Number of selected variants and selection criteria

Scenario	Selection criteria	Selected variants^a^	Accuracy	Slope
Based only on GWAS				
Scenario 1	$50{\text{k}} + {\text{top}}_{{{\text{GWAS}}.{\text{seq}}}}$	3913	0.21 ± 0.01	0.86 ± 0.01
Scenario 2	$50{\text{k}} + {\text{top}}_{{{\text{GWAS}}.{\text{HD}}}}$	226	0.20 ± 0.01	0.88 ± 0.01
Based only on RHM (1 Mbp)				
Scenario 3	$50{\text{k}} + {\text{top}}_{{{\text{RHM}}.{\text{seq}}}}$	26,808	0.21 ± 0.01	0.90 ± 0.01
Based only on RHM (250 kbp)				
Scenario 4	$50{\text{k}} + {\text{top}}_{{{\text{RHM}}.{\text{seq}}}}$	11,507	0.22 ± 0.01	0.91 ± 0.01
Scenario 5	$50{\text{k}} + {\text{top}}_{{{\text{RHM}}.{\text{HD}}}}$	992	0.22 ± 0.01	0.89 ± 0.01
Based on both RHM (250 kbp) and GWAS				
Scenario 6	$50{\text{k}} + {\text{top}}_{{{\text{GWAS}}.{\text{seq}}\left( {{\text{within}}\,{\text{RHM}}} \right)}}$	413	0.25 ± 0.01	0.94 ± 0.01
Scenario 7	$50{\text{k}} + {\text{top}}_{{{\text{GWAS}}.{\text{HD}}\left( {{\text{within}}\,\,{\text{RHM}}} \right)}}$	49	0.23 ± 0.01	0.97 ± 0.01

In each scenario, a GRM from the 50k was fitted with a GRM from the selected variants

The selected variants were ${\text{top}}_{{{\text{GWAS}}.{\text{seq}}}}$: all variants that passed GWAS $- log_{10} \left( p \right)$ threshold of 3, ${\text{top}}_{{{\text{GWAS}}.{\text{HD}}}}$: all HD variants that passed GWAS $- log_{10} \left( p \right)$ threshold of 3, ${\text{top}}_{{{\text{RHM}}.{\text{seq}}}}$: all sequence variants within RHM windows that passed $- log_{10} \left( p \right)$ threshold of 3, ${\text{top}}_{{{\text{RHM}}.{\text{HD}}}}$: all HD variants within RHM windows that passed $- log_{10} \left( p \right)$ threshold of 3, ${\text{top}}_{{{\text{GWAS}}.{\text{seq}}\left( {{\text{within}}\,{\text{RHM}}} \right)}}$: only sequence variants that passed GWAS $- log_{10} \left( p \right)$ threshold of 3 in RHM windows with $- log_{10} \left( p \right) \ge 3$, ${\text{top}}_{{{\text{GWAS}}.{\text{HD}}\left( {{\text{within}}\,{\text{RHM}}} \right)}}$: only HD variants that passed GWAS $- log_{10} \left( p \right)$ threshold of 3 in RHM windows with $- log_{10} \left( p \right) \ge 3$

^a^Number of selected variants after LD pruning

There was no difference in prediction accuracy when variants were selected from WGS genotypes compared to those selected from HD genotypes when selection was based only on RHM results. However, when selection was based on GWAS results within the identified RHM regions, the prediction accuracy for variants selected from WGS data (scenario 6) slightly improved by 2% compared to those selected from HD data (scenario 7). The highest prediction accuracy was obtained when sequence variants, that passed the GWAS $- log_{10} \left( p \right)$ threshold of 3 within windows identified by RHM (0.25 $\pm 0.01$), were selected. All scenarios showed some degree of bias, with slopes ranging from 0.86 for scenario 1 to 0.97 (nearly unbiased) for scenario 7.

### Variance explained by genome-wide variants and selected variants

**Fig. 9:**
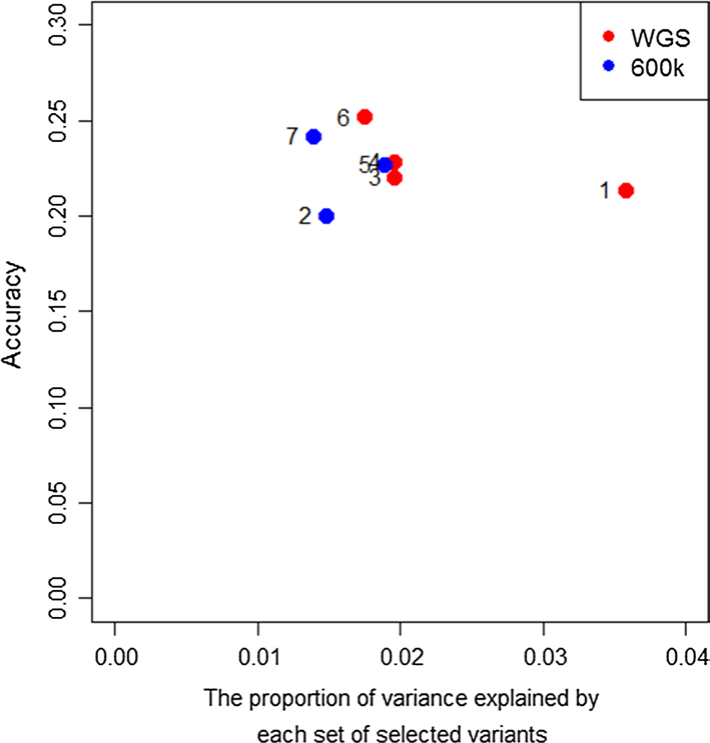
The proportion of phenotypic variance explained by each set of selected markers. Each coloured dot refers to a scenario with its corresponding number. Red dots represent scenarios using WGS data and blue dots represent scenarios using HD data. The x-axis represents the proportion of variance explained by each set of selected variants and the y-axis represents the prediction accuracy obtained when the selected variants were used for genomic prediction

The estimated heritability of WEC, based on pedigree or WGS data, was moderate (0.20 ± 0.03), which suggests that a reasonable part of the phenotypic variation is heritable and therefore repeatable. $h_{top}^{2}$ varied substantially across the scenarios, ranging from 0.0139 ± 0.0072 for scenario 7 to 0.036 ± 0.013 for scenario 1, and it was generally higher when variants were selected from WGS than HD in the same scenario (Fig. 9). However, differences between scenarios were smallest when variant selection was based only on RHM results (scenario 4 vs. scenario 5) and largest when variant selection was based only on GWAS results (scenario 1 vs. scenario 2). When using selected variants only, the highest prediction accuracy 0.018 ± 0.008 of $h_{top}^{2}$ (10% of the genetic variance) was obtained with the set of variants generated by scenario 6. Although variants used in scenario 1 resulted in lower prediction accuracy than variants in scenario 6, they explained the largest $h_{top}^{2}$ (0.036 ± 0.013, 20% of total heritability) among all the scenarios that were tested. These variants probably estimate a proportion of the polygenic effect rather than accurately estimating the genetic variance due to the causal variants only.

## Discussion

In this study, we investigated the possible improvements in QTL discovery and accuracy of genomic prediction for parasite resistance in Australian sheep when using whole-genome sequence data. Using a GRM built from WGS data explained marginally more genetic variation than a GRM built from 50k (4% more) or HD SNP genotypes (1% more). The accuracy of genomic prediction improved by 3 and 1% when using WGS data compared to using 50k and HD genotypes, respectively. The bias of genomic prediction also decreased by 13 and 4% when using WGS data compared to using 50k and HD SNP genotypes, respectively. Fitting variants selected from WGS together with the 50k SNP genotypes improved the prediction accuracy for parasite resistance substantially, up to 9%, compared to fitting the 50k SNPs alone. Of all the scenarios tested, the highest prediction accuracy i.e. $0.25 \pm 0.01$ was reached when variants from WGS were selected based on a GWAS $- log_{10} \left( p \right)$ threshold of 3 in windows identified by RHM.

Significant regions identified by RHM jointly explained only 10% of the estimated heritability. RHM using a window size of 250 kbp narrowed the peaks for those regions although the *p* values remained similar. Using smaller window sizes (e.g. 150 kbp), in an attempt to improve the mapping resolution, did not further narrow those peaks or provide $- log_{10} \left( p \right)$ values higher than 3. In fact, the significance of the peaks decreased with window sizes smaller than 250 kb, except for a slight improvement in the region on OAR2. Overall, the significance level and the size of the QTL effects were relatively small, which combined with the observation that only a small part of the genetic variance was explained by the significant regions, suggests that the genetic architecture underlying the WEC phenotype is relatively polygenic. A similar observation was made by Riggio et al. [24] when using RHM for mapping QTL regions for parasite resistance in Scottish blackface sheep.

In comparison with previous mapping studies, the identified OAR2 region fell within the QTL region (61.7–137.9 Mbp) reported by Crawford et al. [25] for resistance to *T. colubriformis* in Romney and Coopworth crossbred sheep, and partly overlapped with the QTL region (117.9–133.9 Mbp) reported by Davies et al. [26] for resistance to *Nematodirus* in Scottish blackface sheep. The region on OAR6 corresponds to the QTL region (33–39 Mbp) reported by Riggio et al. [24] for resistance to strongyles in Scottish Blackface sheep and the QTL region (25.1–62.6 Mbp) reported by Silva et al. [27] for resistance to *H. contortus* and *Trichostrongylus* in a backcross of Red Maasai and Dorper sheep. The region identified on OAR3 (148–149 Mbp) is close to the *interferon gamma* locus (*IFNG*) at 151 Mbp, which plays a crucial role in the regulation of innate and adaptive immune responses against pathogens [28, 29]. Comparison with other mapping studies showed that this region was within the QTL region (127.3–157.8 Mbp) reported by Dominik et al. [30] for eosinophil count in a Romney × Merino backcross sheep and the QTL region (138.6–150.3 Mbp) reported by Davies et al. [26] for immunoglobulin A (IgA) activity in Scottish blackface sheep. In addition, Riggio et al. [24] identified two significant SNPs around 150 Mbp on OAR3 that were associated with IgA and WEC traits in Scottish blackface sheep.

In this study, increasing marker density from 50k to WGS genotypes resulted only in a small improvement in prediction accuracy for parasite resistance. Similar results were also reported for growth and meat quality traits in the Australian sheep population when increasing the marker density from 50k to WGS genotypes (2% on average) [31, 32]. Although increasing marker density to WGS adds a very large number of genome-wide markers, only a few of these are within or close to causal mutations, leaving the majority of markers in weak or incomplete LD with causal mutations. Variants in weak or incomplete LD with causal mutations add noise, thus limiting the accuracy of genomic prediction, which means that only a limited increase in prediction accuracy will be achieved when using all sequence data unless only the variants in strong LD with causal mutations are used. This agrees with results from simulation studies by van den Berg et al. [9] and Pérez-Enciso et al. [33], which showed a rapid decrease in prediction accuracy when more variants in low LD with causal mutations were included in the prediction models compared to fitting only causal mutations.

In our data, we used individuals from a range of breeds. Using many markers that are not closely linked to QTL would be even less useful in this case, because the LD phases between observed variants and QTL across populations are more inconsistent. LD between markers and QTL would be conserved over shorter distances across-breeds than within-breeds. However, using markers in close LD with causal variants may provide information for genomic prediction across breeds, although to date there is little evidence that this is meaningful across breeds. A multibreed population can be valuable for fine mapping of QTL with WGS data, which provide more predictive markers that are in closer LD with causal variants. In this study, the top variants from WGS data increased prediction accuracy more than those from HD data when fitting the top variants from the identified regions and the 50k markers in a separate GRM. For the same sheep population, Duijvesteijn et al. [34] performed a multi-breed GWAS using WGS data on five meat quality traits and detected many new regions that had not been previously identified using lower density SNP arrays. Moghaddar et al. [32] showed that when fitting sequence variants selected from these identified regions together with the standard 50k variants, prediction accuracy for the meat quality traits improved by up to 6% for Australian sheep.

Fitting a GRM from the selected variants together with a GRM from the 50k SNP genotypes substantially improved the prediction accuracy for parasite resistance compared to fitting only 50k SNP genotypes. In this study, the selected variants were obtained from either the HD or WGS data and are likely to be closer to causal mutations than variants from 50k genotypes. Importantly, when variants were selected from WGS data, the prediction accuracy slightly improved by an additional 2% over HD selected SNPs. This is probably because variants selected from WGS data are more likely to be in high LD with rare causal variants that are not fully tagged by variants selected from the HD SNP panel.

In our study, variants were selected from a separate QTL discovery set and their effects were estimated and validated using the training/validation set. This procedure was performed in order to avoid any potential bias in estimating breeding values [4]. The dataset was split randomly into a QTL discovery set and a training/validation set while simultaneously retaining the same proportion of breeds in each set. The estimates of QTL variants can be biased if they are selected and their effects subsequently estimated and validated using the same dataset. Hence, there is a trade-off such that when including all the animals in the training dataset the prediction accuracy based on the 50k genotypes improves slightly (i.e. prediction accuracy was 0.18 using the larger training dataset compared to 0.16 using the reduced training dataset), however, there is potential for the bias to be quite severe, since we select relatively only a few of the top variants from the larger dataset. This was also observed by [4]. Splitting the data in this way requires to find a balance between the power and accuracy of QTL discovery and the accuracy of genomic prediction although we did not attempt to formally optimise this balance.

The improvement in accuracy of genomic predictions when fitting the selected variants in a separate variance component is most likely due to the GBLUP model with two components of genetic effects, which allows the effects of the selected variants from HD or WGS data to have a larger variance than those from the 50k genotypes, thus putting more weight on the preselected variants. This is in line with results from a study by Brøndum et al. [7] who used variants selected from WGS data by GWAS to predict the genetic merit of production traits in dairy cattle. Their study reported up to 4% increases in prediction accuracy for Nordic Holstein animals, up to 5% for French Holstein, and up to 3% for Nordic Reds when variants selected from WGS data were fitted in a separate component together with the 50k genotypes. Furthermore, van den Berg et al. [8] reported substantial increases in prediction accuracy for milk production in Holstein, Jersey and Danish Red cattle up to 10% when sequence variants selected from multi-breed GWAS were fitted in a separate component with the 50k genotypes, whereas there was no increase in prediction accuracies for Holstein cattle when all sequence variants were used for genomic predictions [3, 4].

GWAS have been increasingly used in genomic prediction studies to identify and select variants to be included in genomic prediction models (e.g. [4, 7, 35]). Unfortunately, type-1 (false positive) and type-2 (false negative) errors can complicate variant selection and obscure the genetic architecture of the trait. In this study, the prediction accuracy improved by 5% when selection was based only on GWAS results across the entire genome, and 9% when selection was limited only to variants that were detected by GWAS in regions identified by RHM. On the one hand, the probability of type-1 errors in GWAS is generally minimized by setting very stringent thresholds [36–38]. These thresholds are reasonable because after testing tens of millions of genome-wide variants, there can be many random variants that have small *p* values purely by chance. However, avoiding type-1 errors may inflate type-2 errors, especially when GWAS are underpowered because of the small effects of causal mutations and/or limited sample size. Balancing between both type-1 and type-2 errors is therefore crucial for GWAS analyses. On the other hand, RHM can increase the power of detection by integrating the effects of multiple variants that are grouped together in a sliding window [13, 24, 39]. Furthermore, RHM can potentially control type-1 errors much better than GWAS because the number of tests generated by RHM is much smaller than that by GWAS, especially when the associations are performed on WGS data (~ 5000 tests from RHM using a window size of 1 Mbp compared to tens of millions of tests from GWAS).

In spite of the potential advantage of using RHM over GWAS in controlling both type-1 and type-2 errors, GWAS can potentially map the causal variants more precisely than RHM, especially when sequence data are used. RHM facilitates the capture of genetic variation in a given region by integrating the effects of all variants, which may also contain variants with no effect on the trait. For better optimization, one might suggest performing RHM with smaller window sizes. However, in this study, this was not always the case. Moving window size from 250 kbp to smaller sizes (e.g. 150 kbp) led to improved mapping power only in a few cases, i.e. for some windows in the region on OAR2 (results not shown). The highest accuracy (0.25 ± 0.01) was obtained when selection within each RHM window was limited to the variants detected from GWAS. This confirms our expectation that restricting the number of prediction variants per window results in higher prediction accuracy than using all the variants. Similar results were also reported by van den Berg et al. [8] who obtained higher prediction accuracies when the number of selected variants per QTL interval was limited to the few variants with the lowest *p* values than selecting all the variants within the QTL region. Our results suggest that RHM combined with GWAS could be a better approach for mapping regions and increasing the prediction accuracy for parasite resistance.

## Conclusions

Our findings show that, by carefully selecting variants from sequence data, the accuracy of genomic predictions can be substantially improved compared to a standard GBLUP based on 50k SNP data. The largest observed gain was a 9% increase in accuracy (from 0.16 to 0.25), which was achieved when selection was restricted to sequence variants detected by GWAS in regions identified by RHM.

## References

[1] Lane J, Jubb T, Shepherd R, Webb-Ware J, Fordyce G. Priority list of endemic diseases for the red meat industries. Final Report. St Lucia: Meat & Livestock Australia L; 2015.

[2] Goddard ME, Hayes BJ, Meuwissen TH (2010). Genomic selection in livestock populations. Genet Res.

[3] Van Binsbergen R, Calus MP, Bink MC, Eeuwijk FA, Schrooten C, Veerkamp RF (2015). Genomic prediction using imputed whole-genome sequence data in Holstein Friesian cattle. Genet Sel Evol.

[4] Veerkamp RF, Bouwman AC, Schrooten C, Calus MP (2016). Genomic prediction using preselected DNA variants from a GWAS with whole-genome sequence data in Holstein-Friesian cattle. Genet Sel Evol.

[5] MacLeod IM, Hayes BJ, Goddard ME (2014). The effects of demography and long term selection on the accuracy of genomic prediction with sequence data. Genetics.

[6] Lee SH, Clark S, van der Werf J (2017). Estimation of genomic prediction accuracy from reference populations with varying degrees of relationship. PLoS One.

[7] Brøndum RF, Su G, Janss L, Sahana G, Guldbrandtsen B, Boichard D (2015). Quantitative trait loci markers derived from whole genome sequence data increases the reliability of genomic prediction. J Dairy Sci.

[8] van den Berg I, Boichard D, Lund MS (2016). Sequence variants selected from a multi-breed GWAS can improve the reliability of genomic predictions in dairy cattle. Genet Sel Evol.

[9] van den Berg I, Boichard D, Guldbrandtsen B, Lund MS (2016). Using sequence variants in linkage disequilibrium with causative mutations to improve across-breed prediction in dairy cattle: a simulation study. G3 (Bethesda).

[10] Bolormaa S, Hayes BJ, van der Werf JH, Pethick D, Goddard ME, Daetwyler HD (2016). Detailed phenotyping identifies genes with pleiotropic effects on body composition. BMC Genomics.

[11] Bolormaa S, Swan AA, Brown DJ, Hatcher S, Moghaddar N, van der Werf JH (2017). Multiple-trait QTL mapping and genomic prediction for wool traits in sheep. Genet Sel Evol.

[12] Bolormaa S, Pryce JE, Reverter A, Zhang Y, Barendse W, Kemper K (2014). Multi-trait, meta-analysis for detecting pleiotropic polymorphisms for stature, fatness and reproduction in beef cattle. PLoS Genet.

[13] Nagamine Y, Pong-Wong R, Navarro P, Vitart V, Hayward C, Rudan I (2012). Localising loci underlying complex trait variation using regional genomic relationship mapping. PLoS One.

[14] van der Werf JHJ, Kinghorn BP, Banks RG (2010). Design and role of an information nucleus in sheep breeding programs. Anim Prod Sci.

[15] Whitlock HV (1948). Some modifications of the McMaster helminth egg-counting technique and apparatus. J Counc Sci Ind Res Aust.

[16] Browning BL, Browning SR (2009). A unified approach to genotype imputation and haplotype-phase inference for large data sets of trios and unrelated individuals. Am J Hum Genet.

[17] Bolormaa S, Chamberlain AJ, Khansefid M, Stothard P, Swan AA, Mason B (2019). Accuracy of imputation to whole-genome sequence in sheep. Genet Sel Evol.

[18] Das S, Forer L, Schönherr S, Sidore C, Locke AE, Kwong A (2016). Next-generation genotype imputation service and methods. Nat Genet.

[19] Loh PR, Danecek P, Palamara PF, Fuchsberger C, Reshef YA, Finucane HK (2016). Reference-based phasing using the Haplotype Reference Consortium panel. Nat Genet.

[20] Daetwyler HD, Brauning R, Chamberlain AJ (2017). 1000 bull genomes and sheepgenomedb projects: enabling cost-effective sequence level analysis globally. Proc Assoc Adv Anim Breed Genet.

[21] Zhou X, Stephens M (2012). Genome-wide efficient mixed-model analysis for association studies. Nat Genet.

[22] Coster A. Package ‘pedigree’. R package version. 2010. http://cran.r-project.org/web/packages/pedigree/pedigree.pdf. Accessed 03 May 2019.

[23] Lee SH, van der Werf J (2016). MTG2: an efficient algorithm for multivariate linear mixed model analysis based on genomic information. Bioinformatics.

[24] Riggio V, Matika O, Pong-Wong R, Stear M, Bishop S (2013). Genome-wide association and regional heritability mapping to identify loci underlying variation in nematode resistance and body weight in Scottish Blackface lambs. Heredity.

[25] Crawford AM, Paterson KA, Dodds KG, Diez Tascon C, Williamson PA, Thomson MR (2006). Discovery of quantitative trait loci for resistance to parasitic nematode infection in sheep: I. Analysis of outcross pedigrees. BMC Genomics.

[26] Davies G, Stear M, Benothman M, Abuagob O, Kerr A, Mitchell S (2006). Quantitative trait loci associated with parasitic infection in Scottish blackface sheep. Heredity.

[27] Silva MV, Sonstegard TS, Hanotte O, Mugambi JM, Garcia JF, Nagda S (2012). Identification of quantitative trait loci affecting resistance to gastrointestinal parasites in a double backcross population of Red Maasai and Dorper sheep. Anim Genet.

[28] Schoenborn JR, Wilson CB (2007). Regulation of interferon-γ during innate and adaptive immune responses. Adv Immunol.

[29] Urban J, Fayer R, Sullivan C, Goldhill J, Shea-Donohue T, Madden K (1996). Local TH1 and TH2 responses to parasitic infection in the intestine: regulation by IFN-gamma and IL-4. Vet Immunol Immunopathol.

[30] Dominik S, Hunt PW, McNally J, Murrell A, Hall A, Purvis IW (2010). Detection of quantitative trait loci for internal parasite resistance in sheep. I. Linkage analysis in a Romney × Merino sheep backcross population. Parasitology.

[31] Moghaddar N, Swan AA, van der Werf JH (2017). Genomic prediction from observed and imputed high-density ovine genotypes. Genet Sel Evol.

[32] Moghaddar N, MacLeod I, Duijvesteijn N, Bolormaa S, Khansefid M, Al-Mamun HA, et al. Genomic evaluation based on selected variants from imputed whole-genome sequence data in Australian sheep populations. In: Proceedings of the 11th World Congress on genetics applied to livestock production: 11–16 February 2018; Auckland; 2018.

[33] Pérez-Enciso M, Rincón JC, Legarra A (2015). Sequence-vs. chip-assisted genomic selection: accurate biological information is advised. Genet Sel Evol.

[34] Duijvesteijn N, Bolormaa S, Gondro C, Clark S, Khansefid M, Moghaddar N, et al. Genome-wide association study of meat quality traits using whole-genome sequence data in a multi-breed sheep population. In: Proceedings of the 11th World Congress on genetics applied to livestock production: 11–16 February 2018; Auckland; 2018.

[35] Ober U, Ayroles JF, Stone EA, Richards S, Zhu D, Gibbs RA (2012). Using whole-genome sequence data to predict quantitative trait phenotypes in *Drosophila melanogaster*. PLoS Genet.

[36] Barsh GS, Copenhaver GP, Gibson G, Williams SM (2012). Guidelines for genome-wide association studies. PLoS Genet.

[37] Johnson RC, Nelson GW, Troyer JL, Lautenberger JA, Kessing BD, Winkler CA (2010). Accounting for multiple comparisons in a genome-wide association study (GWAS). BMC Genomics.

[38] Bush WS, Moore JH (2012). Genome-wide association studies. PLoS Comput Biol.

[39] Riggio V, Pong-Wong R, Sallé G, Usai MG, Casu S, Moreno C (2014). A joint analysis to identify loci underlying variation in nematode resistance in three European sheep populations. J Anim Breed Genet.

